# Croatian genetic heritage: an updated Y-chromosome story

**DOI:** 10.3325/cmj.2022.63.273

**Published:** 2022-06

**Authors:** Dragan Primorac, Vedrana Škaro, Petar Projić, Saša Missoni, Ivana Horjan Zanki, Siniša Merkaš, Jelena Šarac, Natalija Novokmet, Andrea Ledić, Adela Makar, Gordan Lauc, Šimun Anđelinović, Željana Bašić, Ivana Kružić, Marijana Neuberg, Martina Smolić, Robert Smolić, Irena Hrstić, Dragan Trivanović, Rijad Konjhodžić, Lana Salihefendić, Naida Babić Jordamović, Damir Marjanović

**Affiliations:** 1 St. Catherine Hospital, Zagreb, Croatia; 2 School of Medicine, University of Split, Split, Croatia; 3 University Department of Forensic Sciences, University of Split, Split, Croatia; 4 Faculty of Medicine, University of Osijek, Osijek, Croatia; 5 Faculty of Dental Medicine and Health, University of Osijek, Osijek, Croatia; 6 School of Medicine Rijeka, University of Rijeka, Rijeka, Croatia; 7 Eberly College of Science, Pennsylvania State University, University Park, PA, USA; 8 Henry C. Lee College of Criminal Justice and Forensic Sciences, University of New Haven, West Haven, CT, USA; 9 Medical School REGIOMED, Coburg, Germany; 10 The National Forensic Sciences University, Gandhinagar, Gujarat, India; 11 Molecular Anthropology Laboratory, Center for Applied Bioanthropology, Institute for Anthropological Research, Zagreb, Croatia; 12 DNA Laboratory, Genos Ltd, Zagreb, Croatia; 13 Department of Pharmacology, Faculty of Pharmacy and Biochemistry, University of Zagreb, Zagreb, Croatia; 14 Ivan Vučetić Forensic Science Centre, Zagreb, Croatia; 15 University Hospital Center, Split, Croatia; 16 University North, Varaždin, Croatia; 17 General Hospital Pula, Pula, Croatia; 18 Alea Genetic Center, Sarajevo, Bosnia and Herzegovina; 19 Department of Genetics and Bioengineering, International Burch University, Sarajevo, Bosnia and Herzegovina

## Abstract

**Aim:**

To analyze an additional set of Y-chromosome genetic markers to acquire a more detailed insight into the diversity of the Croatian population.

**Methods:**

A total of 518 Yfiler Plus profiles were genotyped. Allele frequencies, haplotype frequencies, and haplotype diversity were calculated by using the STRAF software v. 2.0.4. Genetic distances were quantified by *R*st with AMOVA online tool from the YHRD. The evolutionary history was inferred with the neighbor-joining method of phylogenetic tree construction in the MEGAX software. Whit Athey's Haplogroup Predictor v. 5 was used for additional comparison with regional and other European populations.

**Results:**

A total of 507 haplotypes were used for genetic STR analysis. An interpopulation study on 17 Y-STR markers showed the lowest genetic diversity between the Croatian and Bosnian-Herzegovinian populations and the highest between the Croatian and Irish populations. Additional interpopulation comparison with the original 27 Y-STR markers (for the population with available data) was also performed. A total of 518 haplotypes were used in the determination of haplogroup diversity. Haplogroup I with its sublineage I2a expressed the highest prevalence. The second most prevalent haplogroup was R, with its major sublineage R1a, except for the subpopulation of Hvar, where E1b1b was the second most prevalent haplogroup. Rare haplogroups also confirmed in this study were L, T, and Q. G1 was detected for the first time in the Croatian population.

**Conclusion:**

We obtained a new insight into the differences between examined subpopulations of Croatia and their possible (dis)similarities with neighboring and distant populations.

The Y chromosome ( ∼ 60 Mb) is relatively small and inherited from father to son unchanged (apart from for occasional mutations). Except for the small pseudoautosomal regions (PAR), there is no recombination between the X and Y chromosome (1-3). This is why haplotype inheritance through the male lineage can be tracked and analyzed (2,4-6).

The Y chromosome mostly consists of repetitive sequences (around 50%), which are single-base substitutions, Alu elements, and long interspersed nuclear elements (LINEs). Short tandem repeats (STRs) as repetitive elements are the base of population genetic studies. Their average mutational frequency is ∼ 0.2% per generation (7,8).

Y haplogroup can be defined as a part of the Y-chromosome family related by ancestry and determined by a specific set of Y chromosomal single nucleotide polymorphisms (Y-SNPs). It is important to better understand the demographic processes that shaped modern populations (8,9). The low mutation rate makes Y-SNP markers suitable for the conventional method of Y haplogroup defining.

Y-chromosome haplogroups can also be successfully predicted from Y-STR markers (Y-STR haplotype) by using Y-STR haplogroup predicting tools. Lately, this method has drawn attention due to its effectiveness in terms of labor, time, and costs (10). The haplotype helps us to analyze the influence of genes on disease-related alleles and represents the set of alleles on the same chromosome. On the other hand, major haplogroups (branches of Y-chromosome phylogeny), labeled A-T, reflect the establishment and expansion of major population groups and can indicate the time scale and the route of major migration events (11).

The main aim of this research is to update the information about Croatian Y-chromosome diversity by using additional Y-STR loci to compare new results with the previously published results generated using Y-STR and Y-SNP markers (12-20). A secondary aim was to analyze the genetic structure of five regional subpopulations (with the local centers in Osijek, Pula, Varaždin, Split, and Hvar Island) by identifying the most common haplogroups in these regions. The analysis also included the genetic differences between these five subpopulations and their differences with neighboring countries.

## Materials and methods

For this study, buccal swab samples were obtained from 518 unrelated adult male individuals from five different regions of Croatia: Hvar (n = 104), Osijek (n = 110), Pula (n = 99), Varaždin (n = 100), and Split (n = 105).

DNA was extracted with QIAsymphony instrument by using the QIAsymphony DNA Investigator Kit and protocol (Qiagen, Hilden, Germany). It was quantified on Rotor-Gene Q real-time PCR cycler (Qiagen) by using Q-Rex Software and Investigator Quantiplex Pro RGQ Kit. Twenty-seven Y chromosome STR loci were simultaneously amplified with Yfiler Plus PCR Amplification Kit (Applied Biosystems, Foster City, CA, USA). Amplification was carried out following the manufacturer’s protocol. PCR amplification was performed on Mastercycler^®^ nexus SX1 PCR thermal cycler (Eppendorf AG, Hamburg, Germany) according to the manufacturer’s instructions. PCR-amplified products were separated and detected by using standard protocols for electrophoresis on 3500 Genetic Analyzer (Applied Biosystems). Allele calling was performed with GeneMapper ID-X Software v. 1.4 (Applied Biosystems) by using the custom panel and bin sets.

A total of 507 fully genotyped Y-STR profiles from the present study were submitted to Y Chromosome Haplotype Reference Database (YHRD) with the accession numbers as follows: Hvar (n = 104; YA004742), Osijek (n = 109; YA004743), Pula (n = 94; YA004744), Varaždin (n = 98; YA004746), and Split (n = 102; YA004745).

### Statistical analysis

Allele and haplotype frequencies, the number of alleles and different haplotypes, as well as gene and haplotype diversity were estimated to assess the intrapopulation diversity.

Nei’s formula HD = (1- ∑ *p_i_*^2^)*n/(n-1) was used to calculate haplotype diversity; where n is the sample size and *p_i_* is the *i*^th^ haplotype frequency. Gene diversity was calculated as 1- ∑ *p_i_*^2^, where *p_i_* is the allele frequency. Match probability (MP) was calculated with the formula ∑ *p_i_*^2^, where *p_i_* is the frequency of the *i*^th^ haplotype. Discrimination capacity (DC) was determined by dividing the number of haplotypes by the number of individuals in the population (21,22). STRAF software package v. 2.0.4 was used to calculate allele and haplotype frequencies. The same software was used to calculate gene and haplotype diversity (23,24).

*R_s_*_t_, calculated by AMOVA online tool from the YHRD, was used to quantify genetic distances between groups of men and between populations (25,26). Associated probability values (*P* values) with 10 000 permutations were included for the studied populations. The multidimensional scaling plots (MDS) showing the comparison of population haplotype data from YHRD were generated by using genetic distances.

AMOVA analysis was performed with two population groups. The number of the populations with available data for 27 STR loci was relatively small, especially in the closest Croatian neighborhood. Therefore, the first group was analyzed by comparing the 17 Y-STR loci included in the AmpFLSTR Yfiler PCR Amplification Kit. The second group was analyzed by comparing the whole set of 27 Y-STR loci included in the Yfiler Plus PCR Amplification Kit.

The first group of European populations selected for comparison with the population of Croatia by using 17 Y-STRs included Tiroler Unterland, Austria (n = 547); Antwerpen, Belgium (n = 309); Bosnia and Herzegovina (n = 574); Bulgaria (n = 91); Rostock, Germany (n = 598); Greece (n = 191); Hungary (n = 303); Italy (n = 147); Warsaw, Poland (n = 491); Serbia (n = 567); Albania (n = 315); Czech Republic (n = 109), Estonia (n = 123); Ireland (n = 863); Lithuania (n = 531); North Macedonia (n = 493); Norway (n = 1555); Slovenia (n = 294); Sweden (n = 296); and Ukraine (n = 212).

The second group of the worldwide populations selected for comparison with the population of Croatia included Croatia (n = 507, present study); Slovenia (n = 194); Belgium (n = 160); Hungary (n = 218); Austria (n = 392); Germany (n = 495); Italy (n = 689); North Macedonia (n = 295); Serbia (n = 183); Denmark (n = 177); Ethiopia (n = 290); French Polynesia (n = 81); Ghana (n = 584); India (n = 541); Lithuania (n = 251); Mexico (n = 354); Nigeria (n = 337); Pakistan (n = 280); Poland (n = 612); Russian Federation (n = 958); Saudi Arabia (n = 156); Spain (n = 316;); Switzerland (n = 724); and United Kingdom (n = 115).

Available population data and all related references are included in the YHRD (25,26). The evolutionary history was inferred for both sets of markers by using the neighbor-joining (NJ) method of phylogenetic tree construction (27) in MEGAX (28), whereby the optimal tree is shown.

Y-chromosomal haplogroup prediction with allele frequencies on 518 Yfiler Plus profiles was performed by using Whit Athey's Haplogroup Predictor v. 5, an algorithm based on the Bayesian allele-frequency approach (29,30).

## Results and discussion

A total of 518 haplotypes were detected and used for haplogroup prediction. Eleven haplotypes were considered newly detected microvariants, which required additional analysis for confirmation. Therefore, the remaining 507 haplotypes (the ones without newly detected microvariants) were used for additional statistical analysis. On a sample of fully genotyped 507 Y-STR profiles, 502 different haplotypes were detected, with 497 unique haplotypes and 5 haplotypes appearing twice. In addition, 196 alleles at 27 Y-STR loci were detected (Table 1). The loci with the largest number of detected alleles were the DYS385a/b double locus (DYS385a had 8 alleles and DYS385/b had 10 alleles) and DYS481 (14 detected alleles). The loci with the smallest number of detected alleles (only four alleles each) were DYS393, DYS437, and Y-GATA-H4.

**Table 1:** Allele frequencies for the 27 Y-STR loci in the population of Croatia (n = 518). The study included five Croatian regional subpopulations with the local centers in Osijek, Pula, Varaždin, Split, and Hvar Island. Yfiler Plus PCR Amplification Kit (Applied Biosystems) was used

Allele/Locus	DYF387S1a	DYF387S1b	DYS19	DYS385a	DYS385b	DYS389I	DYS389II	DYS390	DYS391	DYS392	DYS393	DYS437	DYS438	DYS439	DYS448	DYS449	DYS456	DYS458	DYS460	DYS481	DYS518	DYS533	DYS570	DYS576	DYS627	DYS635	YGATAH4
7													0.002														
8																			0.006								
9			0.002						0.020				0.079	0.006					0.097			0.012					
10				0.028					0.485				0.602	0.162					0.584			0.014					0.039
11				0.270	0.006	0.002			0.483	0.862			0.256	0.235					0.262			0.124					0.531
12			0.002	0.028	0.002	0.144			0.010	0.041	0.081		0.057	0.333			0.006		0.051			0.517					0.361
13			0.126	0.114	0.036	0.704			0.002	0.065	0854	0.002	0.004	0.235			0.020	0.008				0.321					0.069
14			0134	0394	0320	0146				0012	0059	0432		0030			0077	0032				0012					
15			0249	0059	0406	0002				0018	0006	0465					0509	0249					0006	0022	0014		
16			0.400	0.085	0.049	0.002				0.002		0.101					0.223	0.231					0.041	0.095	0.077		
17			0.083	0.022	0.075										0.002		0.150	0.331					0.178	0.280	0.136		
18			0.004		0.081										0.008		0.016	0.112					0.391	0.416	0.063		
19					0.018										0.357			0.036					0.217	0.128	0.105		
20					0.008										0.556			0.002		0.004			0.110	0.045	0.306	0.024	
21								0.004							0.073					0.083			0.041	0.010	0.187	0.116	
22								0.069							0.002					0.150			0.016	0.004	0.073	0.235	
23								0.148							0.002					0.178					0.032	0.499	
24								0.527												0.077					0.008	0.103	
25								0.237								0.002				0.063						0.022	
26								0.016								0.002				0.016						0.002	
27							0.004									0.014				0.037							
28							0.075									0.073				0.045							
29							0.178									0.144				0.059							
30							0.329									0.168				0.164							
31							0.321									0.233				0.103							
32							0.087									0.191				0.018							
33							0.004									0.128				0.004							
34	0.018						0.002									0.043											
35	0.144	0.034														0.002					0.002						
36	0.150	0.049																			0.018						
37	0.274	0.089																			0.051						
38	0.353	0.363																			0.124						
39	0.059	0.391																			0.292						
40	0.002	0.063																			0.254						
41		0.012																			0.160						
42																					0.067						
43																					0.028						
44																					0.004						

The haplotype diversity for the studied population was 1.0000 ± 0.0014, with DC of 1.00 and MP of 0.01. Genetic diversity ranged from 0.886 for DYS481 to 0.251 for DYS392. The genetic diversity average across all loci was 0.656. With six detected alleles and the lowest genetic diversity, DYS392 was one of the least polymorphic loci in the studied population. Therefore, it was not surprising that, with a frequency of 0.862, allele 11 at DYS392 was the most common allele (Table 1).

In order to determine additional genetic differences, an interpopulation analysis was done between five regions: Hvar (n = 104), Osijek (n = 109), Pula (n = 94), Split (n = 102), and Varaždin (n = 98). The lowest genetic diversity observed for the population of Hvar was compared with the population of Split (*R*st = 0.0009, *P* = 0.3240). The greatest genetic diversity observed for the population of Hvar was compared with the population of Varaždin (*R*st = 0.0979, *P* < *.0001*), the population of Pula (*R*st = 0.0284, *P* = 0.0042), and the population of Osijek (*R*st = 0.0210, *P* = 0.0097). The lowest genetic diversity observed for the population of Osijek was compared with the population of Split (*R*st = 0.0063, *P* = 0.1199) and the population of Pula (*R*st = 0.0069, *P* = 0.1138). The greatest genetic diversity observed for the population of Osijek was compared with the population of Varaždin (*R*st = 0.0551, *P* <0 .0001) and the population of Hvar (*R*st = 0.0210, *P* = 0.0097). The lowest genetic diversity observed for the population of Pula was compared with the population of Osijek (*R*st = 0.0069, *P* = 0.1138). The greatest genetic diversity observed for the population of Pula was compared with the population of Hvar (*R*st = 0.0284, *P* = 0.0042), the population of Split (*R*st = 0.0180, *P* = 0.0233), and the population of Varaždin (*R*st = 0.0166, *P* = 0.0260). The lowest genetic diversity observed for the population of Split was compared with population of Hvar (*R*st = 0.0009, *P* = 0.3240) and the population of Osijek (*R*st = 0.0063, *P* = 0.1199). The greatest genetic diversity observed for the population of Split was compared with the population of Varaždin (*R*st = 0.0821, *P* < 0.0001) and the population of Pula (*R*st = 0.0180, *P* = 0.0233). The lowest genetic diversity observed for the population of Varaždin was compared with the population of Pula (*R*st = 0.0166, *P* = 0.0260). The greatest genetic diversity observed for the population of Varaždin was compared with the population of Hvar (*R*st = 0.0979, *P* < 0.0001), the population of Split (*R*st = 0.0821, *P* < 0.0001), and the population of Osijek (*R*st = 0.0551, *P* < 0.0001).

In order to compare the studied population with a large number of worldwide published population data, interpopulation analyses were performed by comparing the analyzed population with two groups of countries.

In the first group of populations selected for comparison with the population of Croatia by using reduced 17 Y-STR set of markers included 20 populations. The lowest genetic diversity was observed between the currently analyzed population of Croatia and previously published results for the population of Bosnia and Herzegovina (*R*_st_ = 0.0076, *P* = 0.0002) and the population of Serbia (*R*_st_ = 0.0186,* P* < 0.0001). When compared with the present results, other populations with low genetic diversity values include those from Bulgaria (*R*_st_ = 0.0144,* P* < 0.0001), Ukraine (*R*_st_ = 0.0195, *P* * P* < 0.0001), Slovenia (*R*_st_ = 0.0204, *P* < 0.0001), Hungary (*R*_st_ = 0.0238, *P* < 0.0001), Greece (*R*_st_ = 0.0241, *P* < 0.0001), North Macedonia (*R*_st_ = 0.0375, *P* < 0.0001), Italy (*R*_st_ = 0.0659, *P* < 0.0001), Albania (*R*_st_ = 0.0728, *P* < 0.0001), Czech Republic (*R*_st_ = 0.0767, *P* = 0.000), and Austria (*R*_st_ = 0.0795, *P* < 0.0001). The studied population showed the greatest genetic distance from the populations of Ireland (*R*_st_ = 0.3178,* P* < 0.0001), Estonia (*R*_st_ = 0.1877, *P* < 0.0001), Lithuania (*R*_st_ = 0.1706, *P* < 0.0001), Belgium (*R*_st_ = 0.1429, *P* < 0.0001), Norway (*R*_st_ = 0.1270, *P* < 0.0001), Poland (*R*_st_ = 0.1216, *P* < 0.0001), Sweden (*R*_st_ = 0.1209, *P* < 0.0001), and Germany (*R*_st_ = 0.1036, *P* < 0.0001).

The second group of selected countries used a set of all 27 Y-STR markers. The selection was limited since this is an expanded panel of Y-STR markers, and data are not available for many populations. A comparison of the data on 27 Y-STR markers for the first selected group of the current study and the previously published data for 23 populations showed the lowest genetic diversity of the Croatian population and the population of Serbia (*R_st_* = 0.0097, *P* = 0.0055), and Slovenia (*R_st_* = 0.0297, *P* < 0.0001). Other European populations with low genetic diversity values were the populations from Hungary (*R_st_* = 0.0482, *P* < 0.0001), North Macedonia (*R_st_* = 0.0720, *P* < 0.0001), Russian Federation (*R_st_* = 0.0779, *P* < 0.0001), and Poland (*R_st_* = 0.0905, *P* < 0.0001). A higher genetic distance was observed when the study population was compared with other European and worldwide populations.

**Figure 1:**
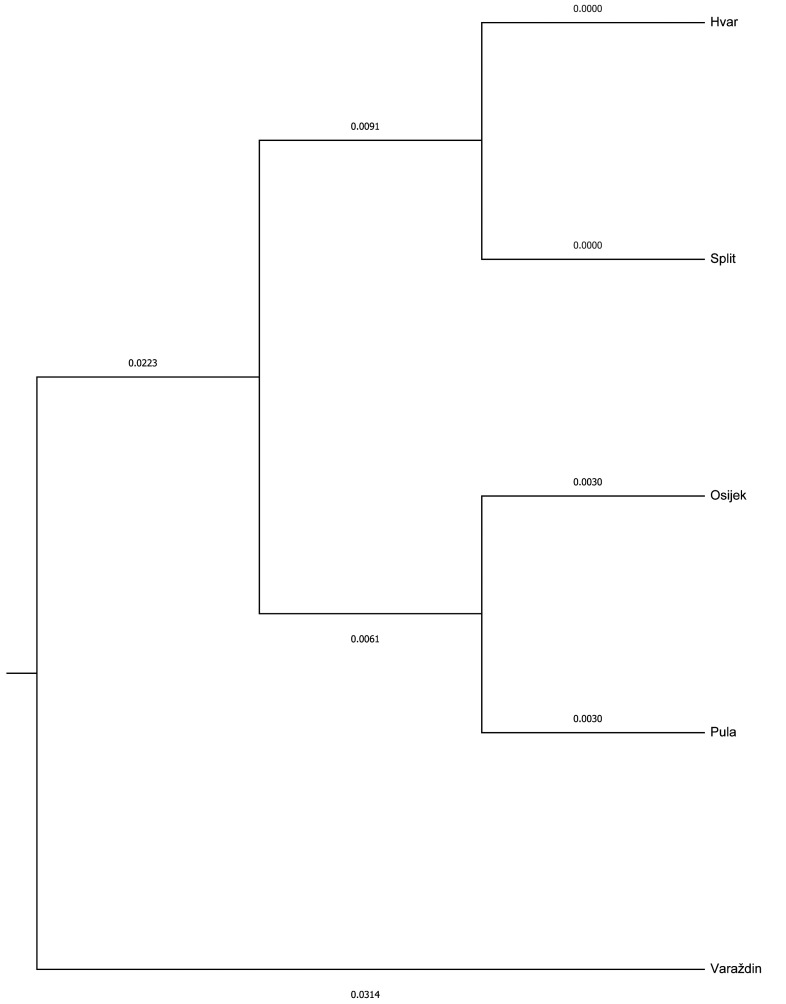
The neighbor-joining phylogenetic tree shows the genetic relationships and clustering between five Croatian regions based on the population study of 27 Y-STR markers (Yfiler Plus PCR Amplification Kit, Applied Biosystems).

To further investigate molecular evolutionary relationships between the geographical subpopulations of Croatia, NJ phylogenetic trees were constructed based on *R*st values for different regions of Croatia (Figure 1).

**Figure 2:**
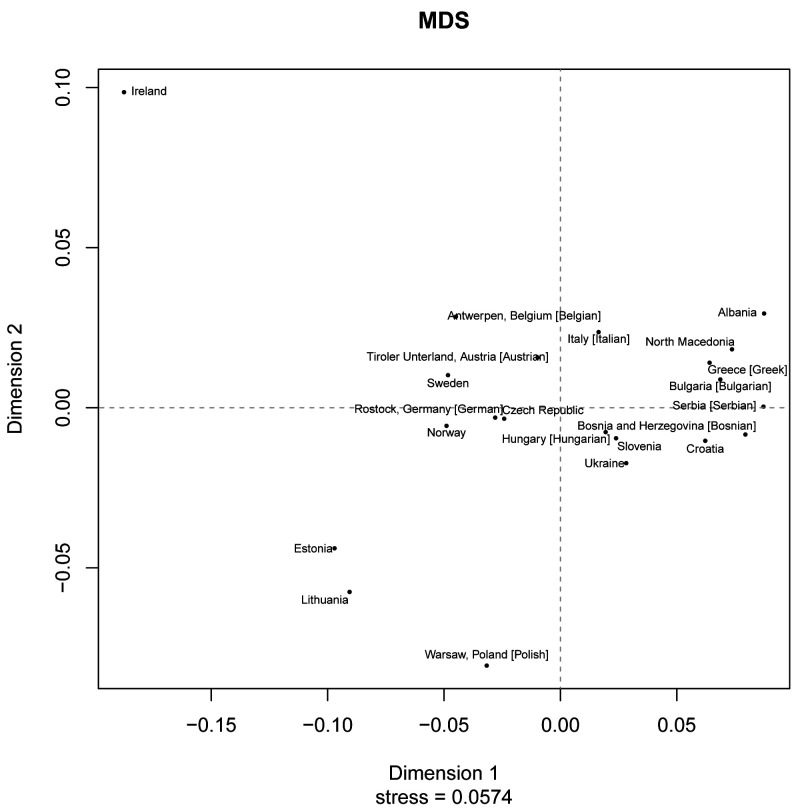
MDS plot showing genetic differentiation between the 21 populations in two dimensions, based on the analysis of available data for 17 Y-STR markers included in the Yfiler marker set (Applied Biosystems).

**Figure 3:**
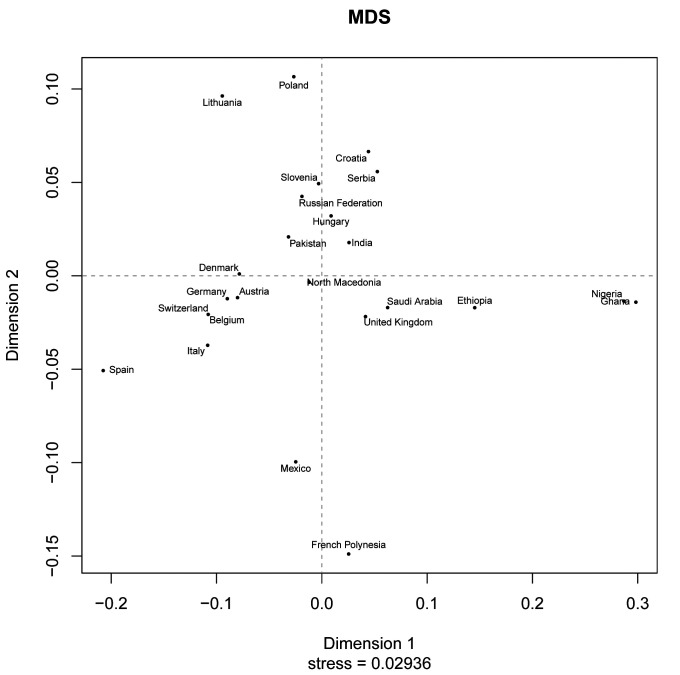
MDS plot showing genetic differentiation between 24 analyzed populations in two dimensions, based on the analysis of available data for 27 Y-STR markers included in the Yfiler Plus PCR Amplification Kit (Applied Biosystems). Data for some neighboring populations (ie, Bosnia and Herzegovina and Montenegro) and a few others that showed clustering when analyzed on 17 Y-STR markers (eg, Bulgaria, Albania, Ukraine etc) were not available and therefore not shown.

Genetic relationships between the investigated populations are shown in MDS plots (Figure 1, Figures 2, and Figure 3). The results of such comparisons confirm the general trends that were shown in Supplementary Table 1(Supplementary Table 1) and Supplementary Table 2(Supplementary Table 2).

The NJ phylogenetic tree shows the genetic relationships and clustering between five Croatian regions based on the population study on 27 Y-STR markers (Figure 1). Hvar and Split subpopulations are clustered together. Osijek and Pula subpopulations are in a separate cluster. The population of the Varaždin region is in a cluster on a different branch, which may indicate its genetic specificity (probably linked with geographical position) relative to the other four examined regional subpopulations.

In the MDS plot showing the group of populations compared by using a set of 17 Y-STR markers, as expected, the Croatian population is clustered with the geographically close populations of Bosnia and Herzegovina, Serbia, and Slovenia. The populations of Ukraine, Bulgaria, and Hungary are also clustered close to these four populations. The next closest European populations are North Macedonia, Albania, and the Czech Republic (Figure 2).

In the MDS plot showing the group of populations compared by using a set of 27 Y-STR markers, the Croatian population is closely clustered with the geographically close populations of Serbia and Slovenia. The populations of Poland, Hungary, and the Russian Federation are also clustered relatively close to these three populations. The next closest European population is that of North Macedonia (Figure 3). The Croatian population was not compared with some neighboring populations (ie, Bosnia and Herzegovina and Montenegro) and a few others that showed clustering when analyzed on 17 Y-STR markers (eg, Bulgaria, Albania, Ukraine, etc) since data on 27 Y-STR markers are unavailable for these populations.

Our study showed a high degree of homogeneity of the Croatian population. Certain genetic similarity was observed at the regional level (between the population of the Pula region and Serbian population; *R*st = 0.0063, *P* = 0.1013), and between the population of the Varaždin region and the neighboring Slovenian population; *R*st = -0.0002, *P* = 0.4124). These results prove again that the Y-chromosome is expected to show greater geographical clustering than other population markers (2,16), but also could potentially mark immigrational impacts from the eastern neighboring countries, such as those in the Istrian region, most probably in the second half of the 20th century. However, these similarities still should be confirmed by additional analysis and increasing/structuring the sample size of the Pula and Varaždin region.

**Figure 4:**
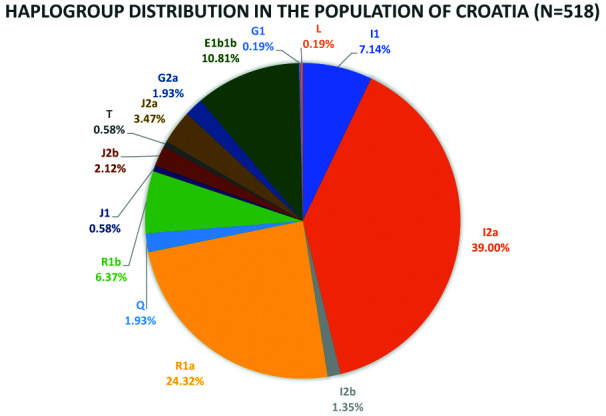
Y-chromosome haplogroup prediction in the Croatian population (n = 518) based on the population study of 27 Y-STR markers (Yfiler Plus PCR Amplification Kit, Applied Biosystems). The study included five Croatian regional subpopulations with the local centers in Osijek, Pula, Varaždin, Split, and Hvar Island.

For the calculation of Y-chromosomal haplogroup prediction and intrapopulation variability between the five subpopulations, 518 Yfiler Plus profiles were used: Hvar (n = 104), Varaždin (n = 100), Split (n = 105), Pula (n = 99), and Osijek (n = 110). Regarding the haplogroup diversity between these five subpopulations, the haplogroup was successfully assigned to all 518 Y-STR profiles (Table 2). The results of Y haplogroup prediction by using Whit Athey's Haplogroup Predictor tool (182) are summarized in Figure 4. Prediction accuracy was 100% in 492 cases. For the remaining 26 samples, the prediction accuracy was 5%. Prediction accuracy varied between 63.1% and 99.58%. Out of 14 detected haplogroups, the most prevalent one was I2a, which accounted for 39% of all samples, followed by R1a (24.32%) and E1b1b (10,18%). The remaining eight haplogroups were less prevalent (Table 2).

**Table 2:** Haplogroup composition in the five regions of Croatia with the local centers in Osijek (n = 110), Pula (n = 99), Varaždin (n = 100), Split (n = 105), and Hvar Island (n = 104). Y chromosome haplogroups prediction is based on the population study of 27 Y-STR markers (Yfiler Plus PCR Amplification Kit, Applied Biosystems)

	Haplogroup/number of haplotypes													
Region	I1	I2a	I2b	J1	J2a	J2b	R1a	R1b	G1	G2a	E1b1b	Q	T	L
Hvar	3	55			4	1	11	4		6	12	8		
Varaždin	8	18	3	1	4	3	38	9			16			
Split	5	53	2		2	3	20	5	1		11	1	2	
Pula	9	31	1	1	3	2	28	8		3	11		1	1
Osijek	12	45	1	1	5	2	29	7		1	6	1		
Total number of haplotypes	37	202	7	3	18	11	126	33	1	10	56	10	3	1

**Figure 5:**
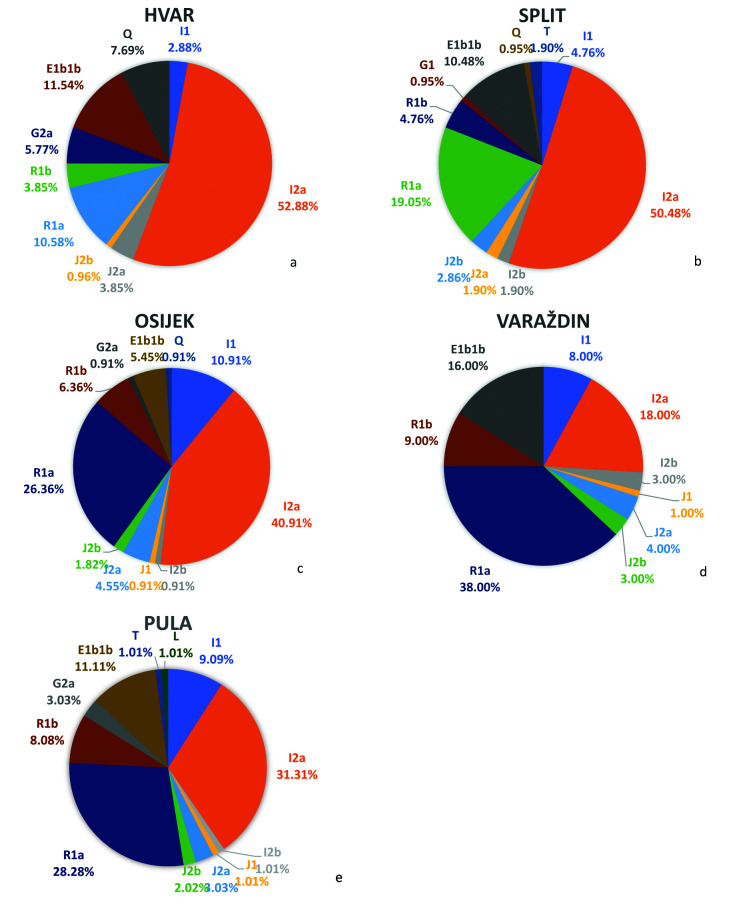
Y-chromosome haplogroup frequency in five Croatian subpopulations: a. Hvar (n = 104), b. Split (n = 105), c. Osijek (n = 110), d. Varaždin (n = 100), e. Pula (n = 99). Y-haplogroup frequencies are determined in the population study of 27 Y-STR markers (Yfiler Plus PCR Amplification Kit, Applied Biosystems).

Four of the five subpopulations of Croatia showed expected results (Figure 5A-C). High frequency of haplogroup I was reported with its known sublineage I2a in the subpopulations: Hvar 52.88%, Split 50.48%, Osijek 40.91%, and Pula 31.31%. Previously published reports demonstrate similar results (12,13,16,18,19). However, slightly different results were obtained in the subpopulation of Varaždin (Figure 5D). In this subpopulation, R1a was the most frequent haplogroup with a frequency of 38%, while the frequency for I2a haplogroup was 18%. Interestingly, R1a was also the dominant haplogroup within the Slovenian population (31), which is the closest neighboring abroad population to Varaždin county. However, as we have already stated, these initially notified similarities still should be confirmed by additional analysis and by increasing/structuring the sample size of the Varaždin region (Figure 5).

In summary, sublineage I2a was generally the most frequent haplogroup in the populations of Croatia in this study, but also in all previously studies (12,13,16,18,19,32). Similar results were obtained in an earlier study, when I-P37 (a former name for the most I2a sublineage) for the Croatian population in Bosnia and Herzegovina was detected at a ratio of 71.1% (15).

Haplogroup I (previously described as Eu7) (12) arrived to the Balkans around 25 000 years ago from the Middle East through Anatolia (9,16). One scenario suggests the possibility of population expansion from one of the post-Glacial refugia into the rest of the Balkan Peninsula (15). There is also a possibility that this haplogroup could be connected with more recent population movements from Eastern Europe, but this idea still has to be examined (9). Definitively, when compared with the other populations in Europe, the I2a haplogroup sublineage is considered a characteristic Southeast European haplogroup (33).

The R1a (previously described as Eu19) (12), as a leading sublineage of haplogroup R, was the second most frequent haplogroup in the studied population of Croatia, with an overall frequency of 24.32%. The following prevalences of haplogroup R1a in the subpopulations of Croatia were reported: Varaždin 38%, Pula 28.28%, Osijek 26.36%, and Split 19.05%. In the subpopulation of Hvar, a small genetic deviation in the frequency of haplogroups R1a and E1b1b was reported. The R1a haplogroup accounted for 10.58%, just slightly lower than haplogroup E1b1b with the frequency of 11.54% (Figure 5A). This is most likely due to the founder effect, which is expected for island populations. In previously reported studies on the mainland population of Croatia, haplogroup R was reported as the second most frequent (13,18,32). Migration theories of R1a origins indicate the outflow of haplogroup R from West Asia to the Balkans as a post-last glacial maximum (LGM) event during the Mesolithic (16,34).

Sublineage R1b (previously described as Eu18) (12) showed a lower frequency in the studied population. The overall frequency of the R1b sublineage for the population of Croatia amounts to 6.37%. The highest frequency of R1b haplogroup was reported in the subpopulation of Varaždin, with a prevalence of 9%, and in Pula, with a frequency of 8.08%. The most similar results were obtained in the Bosnian population based on 481 Y-STR profiles, whereby R1b accounted for 8.75% of the samples (10).

Sublineage E1b1b (previously predominantly described as Eu4) (12) is the most frequent “neolithic haplogroup” for men in this part of Europe (16). In the present study, E1b1b was detected with a frequency of 10.81%. The highest prevalence of this haplogroup was reported in the subpopulation of Varaždin, with a frequency of 16%. In the other four subpopulations, the frequencies were as follows: Hvar 11.54%, Pula 11.11%, Split 10.48%, and Osijek 5.45%. According to the recently published results, this haplogroup is slightly less frequent than in the closest neighboring population of Bosnia and Herzegovina (14.58%) (10). There are two theories about E1b1b arrival in Europe. One theory is a post-LGM event from Asia and Africa during the Neolithic period, while the other theory suggests that this haplogroup is Balkan-specific, and originated around 8000 years ago during Greek colonization in the northern part of the Peninsula (16,35). This ancient European haplogroup shows its possible dual origin from two different source populations, during the recolonization of Europe from Iberia and from West Asia (16,32).

An approximate comparison between the frequencies of the earlier used Y chromosome lineage (Eu) determined by Semino et al (12) and the frequency of the currently used haplogroups detected in the Croatian population is shown in Table 3. The exact comparison is not possible because current nomenclature offers a more detailed and precise insight into Y chromosome diversity. However, this table could approximate a comparison between early and currently detected Y chromosome diversity within the Croatian population.

**Table 3:** An approximate comparison between the frequency of the earlier used Y chromosome lineage (Eu) determined by Semino et al (12) and the frequency of the currently used haplogroups detected in the Croatian population*

EU	Current Hg	Closest Joint Mutation	Frequencies (%)	
**Semino et al (year 2000)**	**Current Croatian data (year 2022)**
EU 4	E1b1b	M35	6.9	10.8
EU 7	I	M170	44.8	47.49
EU 18	R1b	M173	10.3	6.37
EU 19	R1a	M17	29.3	24.32

*The exact comparison is not possible because the current nomenclature offers more detailed and precise insight into Y-chromosome diversity.

Rare haplogroups discovered in this study were Q, T, L and G1, each present in 1.93%, 0.58%, 0.19%, and 0.19% of all samples, respectively. Haplogroup L is associated with South Asia and India but is also found in low frequencies in Central Asia, Southwest Asia, and Southern Europe. With its alternative phylogenetic name K1a, haplogroup L is closely related to haplogroup T (36). Haplogroup T (phylogenetic name K1b) originates from Western Asia, spreading to East Africa, South Asia, and Southern Europe (37,38). Haplogroup Q is the only Pan-American haplogroup and confirms the Asian origin of Native Americans (39). It provides insight into the main Asian-American migrations. We detected haplogroup G1 for the first time within the Croatian population. This haplogroup is found predominantly in the Eurasian population, particularly in Iran, and is very rare in Europe. Some authors suggest that this rare haplogroup could have been related to the expansion of Iranian speakers northwards to the Eurasian steppe (40). However, its origin is still not clearly described.

This study provided a more detailed insight into the genetic diversity of the subpopulations of Croatia. Furthermore, our results generally confirmed previous results. *R*st statistics was used to compare 27 Y-STR loci data from the studied Croatian population with data of other populations available from YHRD. The results indicate that the Croatian population does not deviate significantly from the neighboring populations of Bosnia and Herzegovina, Slovenia, and Serbia. This proves that the Y chromosome genetic marker has a noticeable geographical background (2,15), and this analysis resulted in expected geographic clustering.

Most of the Croatian men (“owners” of HgI, R1a, and R1b) harbor the ancestral genetic impact of Old European people who settled in Europe approximately 25 000-30 000 years ago and survived the LGM in several different refugia (16). Our results on new additional Y-STR loci confirmed that more than 78% of the contemporary Croatians are included in that group. The rest of the population relates to the people who arrived mostly during the Neolithization process. A small portion of the examined population originated from the “owners” of rare haplogroups in terms of European genetic diversity, the origin of which is still not clarified. The use of additional Y-STR loci provided detailed insight and supplementary information regarding genetic diversity of the Croatian population.

The limitation of the study is that some of the regional populations could not be compared with the Croatian population due to the lack of published data. Therefore, two levels of the comparison were necessary. Furthermore, current results for Pula and Varaždin subpopulations indicate somewhat greater similarity to regional populations. Although this could be explained based on historical facts, more detailed structuring of these populations should be performed to confirm the obtained results.
