# Supplementary material for: Croatian genetic heritage: an updated Y-chromosome story
Source: Croat Med J. 2022 Jun;63(3):273–86. doi: 10.3325/cmj.2022.63.273 (PMC9284021; doi:10.3325/cmj.2022.63.273)
Supplement: Supplementary Table 1 [file CroatMedJ_63_s020.pdf]

Supplementary Table 1. Interpopulation comparison over 17 Y-STR loci (included in the Yfiler™ marker set, Applied Biosystems) of the current data with 20 previously published European populations using genetic distance *R*st values and *P* values. Data was obtained from the relevant publications and accessed through the YHRD.

**Population**	Croatia	Austria	Belgium	Bosnia and Herzegovina	Bulgaria	Germany	Greece	Hungary	Italy	Poland	Serbia	Albania	Czech Republic	Estonia	Ireland	Lithuania	North Macedonia	Norway	Slovenia	Sweden	Ukraine
Croatia	-	0.0000	0.0000	0.0002	0.0089	0.0000	0.0000	0.0000	0.0000	0.0000	0.0000	0.0000	0.0000	0.0000	0.0000	0.0000	0.0000	0.0000	0.0000	0.0000	0.0001
Austria	0.0795	-	0.0000	0.0000	0.0000	0.0000	0.0000	0.0000	0.0035	0.0000	0.0000	0.0000	0.0079	0.0000	0.0000	0.0000	0.0000	0.0000	0.0000	0.0000	0.0000
Belgium	0.1429	0.0188	-	0.0000	0.0000	0.0000	0.0000	0.0000	0.0000	0.0000	0.0000	0.0000	0.0007	0.0000	0.0000	0.0000	0.0000	0.0000	0.0000	0.0000	0.0000
Bosnia and Herzegovina	0.0076	0.0988	0.1671	-	0.0034	0.0000	0.0000	0.0000	0.0000	0.0000	0.0002	0.0000	0.0000	0.0000	0.0000	0.0000	0.0000	0.0000	0.0000	0.0000	0.0000
Bulgaria	0.0144	0.0649	0.1467	0.0186	-	0.0000	0.1307	0.0001	0.0000	0.0000	0.0095	0.0041	0.0000	0.0000	0.0000	0.0000	0.0706	0.0000	0.0001	0.0000	0.0003
Germany	0.1036	0.0214	0.0173	0.1382	0.1206	-	0.0000	0.0000	0.0000	0.0000	0.0000	0.0000	0.2638	0.0000	0.0000	0.0000	0.0000	0.0000	0.0000	0.0000	0.0000
Greece	0.0241	0.0519	0.1187	0.0269	0.0048	0.1025	-	0.0001	0.0008	0.0000	0.0001	0.0024	0.0000	0.0000	0.0000	0.0000	0.0785	0.0000	0.0000	0.0000	0.0000
Hungary	0.0238	0.0252	0.0623	0.0451	0.0354	0.0321	0.0283	-	0.0001	0.0000	0.0000	0.0000	0.0024	0.0000	0.0000	0.0000	0.0000	0.0000	0.0973	0.0000	0.0130
Italy	0.0659	0.0144	0.0441	0.0744	0.0465	0.0546	0.0223	0.0273	-	0.0000	0.0000	0.0000	0.0000	0.0000	0.0000	0.0000	0.0000	0.0000	0.0000	0.0000	0.0000
Poland	0.1216	0.1049	0.1305	0.1687	0.1603	0.0580	0.1555	0.0679	0.1542	-	0.0000	0.0000	0.0000	0.0000	0.0000	0.0000	0.0000	0.0000	0.0000	0.0000	0.0000
Serbia	0.0186	0.1087	0.1870	0.0068	0.0145	0.1612	0.0182	0.0612	0.0735	0.2021	-	0.0000	0.0000	0.0000	0.0000	0.0000	0.0000	0.0000	0.0000	0.0000	0.0000
Albania	0.0728	0.0960	0.1714	0.0585	0.0258	0.1723	0.0159	0.0865	0.0527	0.2372	0.0336	-	0.0000	0.0000	0.0000	0.0000	0.0112	0.0000	0.0000	0.0000	0.0000
Czech Republic	0.0767	0.0156	0.0252	0.1086	0.0875	0.0012	0.0704	0.0176	0.0390	0.0519	0.1330	0.1324	-	0.0000	0.0000	0.0000	0.0000	0.0018	0.0002	0.0000	0.0000
Estonia	0.1877	0.1038	0.0940	0.2198	0.2054	0.0623	0.1630	0.1043	0.1226	0.1052	0.2477	0.2263	0.0693	-	0.0000	0.0080	0.0000	0.0000	0.0000	0.0000	0.0000
Ireland	0.3178	0.1360	0.0852	0.3403	0.3446	0.1221	0.3169	0.2276	0.2137	0.2475	0.3677	0.3590	0.1671	0.2125	-	0.0000	0.0000	0.0000	0.0000	0.0000	0.0000
Lithuania	0.1706	0.1162	0.1109	0.2114	0.2008	0.0615	0.1754	0.0977	0.1484	0.0555	0.2415	0.2548	0.0652	0.0121	0.2139	-	0.0000	0.0000	0.0000	0.0000	0.0000
North Macedonia	0.0375	0.0747	0.1458	0.0312	0.0067	0.1358	0.0036	0.0528	0.0419	0.1887	0.0165	0.0067	0.1028	0.2029	0.3271	0.2178	-	0.0000	0.0000	0.0000	0.0000
Norway	0.1270	0.0391	0.0394	0.1616	0.1473	0.0160	0.1183	0.0500	0.0715	0.0862	0.1797	0.1913	0.0178	0.0553	0.1700	0.0670	0.1535	-	0.0000	0.0000	0.0000
Slovenia	0.0204	0.0376	0.0887	0.0463	0.0297	0.0466	0.0284	0.0025	0.0439	0.0639	0.0606	0.0868	0.0269	0.1196	0.2641	0.1046	0.0511	0.0589	-	0.0000	0.0281
Sweden	0.1209	0.0427	0.0447	0.1437	0.1301	0.0400	0.0894	0.0545	0.0449	0.1360	0.1546	0.1406	0.0363	0.0646	0.2141	0.0956	0.1176	0.0147	0.0674	-	0.0000
Ukraine	0.0195	0.0603	0.1135	0.0461	0.0405	0.0599	0.0384	0.0079	0.0619	0.0530	0.0640	0.1005	0.0396	0.1254	0.2892	0.1021	0.0632	0.0845	0.0060	0.0950	-
