# Supplementary material for: Croatian genetic heritage: an updated Y-chromosome story
Source: Croat Med J. 2022 Jun;63(3):273–86. doi: 10.3325/cmj.2022.63.273 (PMC9284021; doi:10.3325/cmj.2022.63.273)
Supplement: Supplementary Table 2 [file CroatMedJ_63_s021.pdf]

Supplementary Table 2. Interpopulation comparison over 27 Y-STR loci (included in the Yfiler™ Plus PCR Amplification Kit, Applied Biosystems) of the current data with 23 previously published Worldwide populations using genetic distance *R*st values and *P* values. Data was obtained from the relevant publications and accessed through the YHRD.

**Population**	Croatia	Austria	Belgium	Denmark	Ethiopia	French Polynesia	Germany	Ghana	Hungary	India	Italy	Lithuania	Mexico	Nigeria	North Macedonia	Pakistan	Poland	Russian Federation	Saudi Arabia	Serbia	Slovenia	Spain	Switzerland	United Kingdom
Croatia	-	0.0000	0.0000	0.0000	0.0000	0.0000	0.0000	0.0000	0.0000	0.0000	0.0000	0.0000	0.0000	0.0000	0.0000	0.0000	0.0000	0.0000	0.0000	0.0055	0.0000	0.0000	0.0000	0.0000
Austria	0.1255	-	0.0000	0.0024	0.0000	0.0000	0.0052	0.0000	0.0000	0.0000	0.0000	0.0000	0.0000	0.0000	0.0000	0.0000	0.0000	0.0000	0.0000	0.0000	0.0000	0.0000	0.0000	0.0000
Belgium	0.2031	0.0197	-	0.0000	0.0000	0.0000	0.0027	0.0000	0.0000	0.0000	0.0000	0.0000	0.0000	0.0000	0.0000	0.0000	0.0000	0.0000	0.0000	0.0000	0.0000	0.0000	0.4444	0.0000
Denmark	0.1290	0.0099	0.0412	-	0.0000	0.0000	0.0013	0.0000	0.0000	0.0000	0.0000	0.0000	0.0000	0.0000	0.0000	0.0000	0.0000	0.0000	0.0000	0.0000	0.0000	0.0000	0.0000	0.0000
Ethiopia	0.1704	0.2165	0.2801	0.2495	-	0.0000	0.0000	0.0000	0.0000	0.0000	0.0000	0.0000	0.0000	0.0000	0.0000	0.0000	0.0000	0.0000	0.0000	0.0000	0.0000	0.0000	0.0000	0.0000
French Polynesia	0.1878	0.1419	0.2024	0.1934	0.2763	-	0.0000	0.0000	0.0000	0.0000	0.0000	0.0000	0.0000	0.0000	0.0000	0.0000	0.0000	0.0000	0.0000	0.0000	0.0000	0.0000	0.0000	0.0000
Germany	0.1461	0.0045	0.0104	0.0102	0.2501	0.1709	-	0.0000	0.0000	0.0000	0.0000	0.0000	0.0000	0.0000	0.0000	0.0000	0.0000	0.0000	0.0000	0.0000	0.0000	0.0000	0.0000	0.0000
Ghana	0.2787	0.3710	0.4691	0.4133	0.2459	0.3321	0.4094	-	0.0000	0.0000	0.0000	0.0000	0.0000	0.0012	0.0000	0.0000	0.0000	0.0000	0.0000	0.0000	0.0000	0.0000	0.0000	0.0000
Hungary	0.0482	0.0757	0.1484	0.0879	0.1303	0.1706	0.0947	0.3044	-	0.0000	0.0000	0.0000	0.0000	0.0000	0.0000	0.0000	0.0000	0.0000	0.0000	0.0000	0.0004	0.0000	0.0000	0.0000
India	0.0961	0.1023	0.1642	0.1154	0.1454	0.1681	0.1217	0.2779	0.0201	-	0.0000	0.0000	0.0000	0.0000	0.0000	0.0000	0.0000	0.0000	0.0000	0.0000	0.0000	0.0000	0.0000	0.0000
Italy	0.2043	0.0249	0.0159	0.0575	0.2656	0.1578	0.0281	0.4213	0.1339	0.1529	-	0.0000	0.0000	0.0000	0.0000	0.0000	0.0000	0.0000	0.0000	0.0000	0.0000	0.0000	0.0000	0.0000
Lithuania	0.1914	0.1028	0.1330	0.0847	0.2836	0.2703	0.0922	0.4656	0.1106	0.1226	0.1454	-	0.0000	0.0000	0.0000	0.0000	0.0000	0.0000	0.0000	0.0000	0.0000	0.0000	0.0000	0.0000
Mexico	0.1785	0.0949	0.1173	0.1287	0.2192	0.1469	0.1094	0.3530	0.1398	0.1410	0.1291	0.2279	-	0.0000	0.0000	0.0000	0.0000	0.0000	0.0000	0.0000	0.0000	0.0000	0.0000	0.0000
Nigeria	0.2414	0.3309	0.4230	0.3709	0.2023	0.2829	0.3721	0.0054	0.2563	0.2394	0.3853	0.4213	0.3107	-	0.0000	0.0000	0.0000	0.0000	0.0000	0.0000	0.0000	0.0000	0.0000	0.0000
North Macedonia	0.0720	0.0374	0.0848	0.0627	0.1212	0.1627	0.0548	0.3156	0.0348	0.0656	0.0780	0.1377	0.1012	0.2687	-	0.0000	0.0000	0.0000	0.0000	0.0000	0.0000	0.0000	0.0000	0.0000
Pakistan	0.0854	0.0531	0.1030	0.0519	0.2216	0.1213	0.0644	0.3449	0.0381	0.0503	0.1003	0.0963	0.1256	0.2998	0.0718	-	0.0000	0.0000	0.0000	0.0000	0.0000	0.0000	0.0000	0.0000
Poland	0.0905	0.1294	0.1886	0.1076	0.2569	0.2681	0.1225	0.4045	0.0687	0.0972	0.2007	0.0684	0.2363	0.3703	0.1198	0.0762	-	0.0000	0.0000	0.0000	0.0000	0.0000	0.0000	0.0000
Russian Federation	0.0779	0.0677	0.1188	0.0648	0.1586	0.1833	0.0794	0.2947	0.0303	0.0593	0.1229	0.0704	0.1350	0.2609	0.0471	0.0689	0.0635	-	0.0000	0.0000	0.0000	0.0000	0.0000	0.0000
Saudi Arabia	0.0990	0.1150	0.1891	0.1464	0.0732	0.1506	0.1523	0.2084	0.0562	0.0604	0.1695	0.2070	0.0956	0.1639	0.0577	0.1029	0.1896	0.0794	-	0.0000	0.0000	0.0000	0.0000	0.0002
Serbia	0.0097	0.1259	0.2101	0.1415	0.1231	0.2006	0.1525	0.2604	0.0449	0.0847	0.2057	0.2023	0.1647	0.2159	0.0517	0.1014	0.1189	0.0743	0.0669	-	0.0000	0.0000	0.0000	0.0000
Slovenia	0.0297	0.0671	0.1355	0.0615	0.1703	0.1821	0.0781	0.3378	0.0142	0.0514	0.1387	0.0932	0.1533	0.2887	0.0418	0.0334	0.0310	0.0275	0.0899	0.0423	-	0.0000	0.0000	0.0000
Spain	0.3283	0.1183	0.0490	0.1695	0.4077	0.3475	0.0887	0.5812	0.2931	0.2865	0.0760	0.2378	0.2156	0.5509	0.2032	0.2321	0.2990	0.2268	0.3483	0.3590	0.2795	-	0.0000	0.0000
Switzerland	0.1999	0.0138	-0.0003	0.0299	0.2845	0.1833	0.0075	0.4310	0.1421	0.1632	0.0180	0.1283	0.1143	0.3997	0.0840	0.1026	0.1816	0.1170	0.1850	0.2052	0.1314	0.0581	-	0.0000
United Kingdom	0.1122	0.0813	0.1442	0.1122	0.0780	0.1429	0.1167	0.2236	0.0562	0.0558	0.1344	0.1718	0.0994	0.1752	0.0438	0.0865	0.1744	0.0756	0.0181	0.0808	0.0809	0.3041	0.1438	-
